# Effect of prophylactic antiviral intervention on T cell immunity in hepatitis B virus-infected pregnant women

**DOI:** 10.1186/s12884-023-05700-8

**Published:** 2023-05-27

**Authors:** Meiting Huang, Yunfei Gao, Dandan Liao, Yanchen Ma, Jinna Li, Bo Tang, Yaohua Hao, Xuelian Zhang, Shimin Yin, Xiaohuan Jiang, Jialin Li, Xueru Yin, Yongyin Li, Jing Hu, Zhihua Liu

**Affiliations:** 1 Department of Infectious Diseases, Nanfang Hospital, Southern Medical University, Guangzhou, 510515 China; 2 Department of Obstetrics and Gynaecology, Nanfang Hospital, Southern Medical University, Guangzhou, 510515 China; 3 Department of Obstetrics and Gynaecology, Zengcheng Branch of Nanfang Hospital, Southern Medical University, Guangzhou, 511340 China; 4 Department of Nosocomial Infection Administration, Zhujiang Hospital, Southern Medical University, Guangzhou, 510280 China

**Keywords:** Hepatitis B virus, Pregnant women, Antiviral intervention, Regulatory T cell, T helper cell

## Abstract

**Background:**

Antiviral intervention in hepatitis B virus (HBV)-infected pregnant women can effectively reduce mother-to-child transmission. However, the immunological characteristics of pregnant women with chronic HBV infection and the effects of antiviral intervention during pregnancy on maternal immune response remain unknown. We aimed to investigate these effects by comparing mothers who received antiviral intervention during pregnancy with those who did not.

**Methods:**

Pregnant women positive for hepatitis B surface antigen and hepatitis B e-antigen (HBsAg^+^ HBeAg^+^) were enrolled at delivery, including 34 received prophylactic antiviral intervention during pregnancy (AVI mothers) and 15 did not (NAVI mothers). T lymphocyte phenotypes and functions were analysed using flow cytometry.

**Results:**

At delivery, maternal regulatory T cell (Treg) frequency in AVI mothers was significantly higher than that in NAVI mothers (*P* < 0.002), and CD4^+^ T cells in AVI mothers displayed a decreased ability to secrete IFN-γ (*P* = 0.005) and IL-21 (*P* = 0.043), but an increased ability to secrete IL-10 and IL-4 (*P* = 0.040 and *P* = 0.036), which represented a higher Treg frequency, enhanced Th2 response and suppressed Th1 response. Treg frequency among AVI mothers was correlated negatively with serum HBsAg and HBeAg levels. After delivery, the ability of CD4^+^ T cells or CD8^+^ T cells to secrete IFN-γ or IL-10 was similar and no significant difference in Treg frequency was found between the two groups.

**Conclusions:**

Prophylactic antiviral intervention during pregnancy has an effect on T cell immunity in pregnant women, which was characterised by increased maternal Treg frequency, enhanced Th2 response and suppressed Th1 response at delivery.

**Supplementary Information:**

The online version contains supplementary material available at 10.1186/s12884-023-05700-8.

## Background

Chronic hepatitis B virus (HBV) infection remains a major public health concern. More than 250 million people worldwide are infected with HBV. Of these, 30–50% are attributed to HBV mother-to-child transmission (MTCT), and approximately 887,000 people die each year due to HBV-related diseases [1–4]. In highly endemic areas, approximately 90% of HBV infections during infancy and early childhood through MTCT become chronic, whereas transmission between adults mainly leads to acute infection, with less than 5% developing into chronic cases [5–7]. Therefore, prevention of MTCT potentially represents the most effective way to reduce the global burden of HBV infection. The administration of hepatitis B immunoglobulin and HBV vaccine has reduced MTCT incidence from 90% to 5–10% [7–10], and immunoprophylaxis mainly fails in infants born to HBeAg-positive mothers with high viral load of HBV [11–13]. In the past decade, numerous clinical studies have demonstrated that prophylactic antiviral intervention during pregnancy in HBV-infected mothers was a safe and effective measure for preventing MTCT [14–17]. In the hepatitis B guidelines of the American Association for the Study of Liver Diseases (AASLD), the European Association for the Study of the Liver (EASL), the Asian Pacific Association for the Study of the Liver (APASL), and management algorithm for MTCT [18–21], it is recommended that HBV-infected pregnant women with high levels of HBV DNA (> 2 × 10^5^–2 × 10^6^ IU/mL) should receive antiviral therapy during late pregnancy to reduce MTCT risk. In a large cohort study in China, it was reported that 49.3% of pregnant women with HBV infection have received antiviral intervention during pregnancy to interrupt MTCT in recent years [22].

A series of changes in hormones and other components of the immune system occur during pregnancy, due to tolerance of foetal semi-allogeneic antigens and foetus development [23, 24]. In various stages of pregnancy, the balance between pro-inflammatory and anti-inflammatory of pregnant women is dynamically modulated. The early pregnancy is predominantly pro-inflammatory stage, with increase in Th1 cytokines. Then, anti-inflammatory immunity featured in Th2 bias becomes predominance in the middle pregnancy, while pro-inflammatory cytokine milieu once again dominates in the late pregnancy [25]. In the special period of pregnancy, the dynamic immunity of mothers may have influence on HBV infection. Previous studies have shown that antiviral therapy with nucleot(s)ide analogues (NAs) could impact on T cell immunity of chronic hepatitis B patients [26]. Recently, our team found that T cells in mothers with ALT flare postpartum produced more pro-inflammatory cytokines, and less anti-inflammatory cytokines than those in mothers without ALT flare postpartum [27]. However, the effect of use of antiviral drugs during pregnancy on mother’s T cell immunity is still unknown. To provide evidence for managing chronic HBV-infected pregnant women, it is needed to explore alteration of T cell immunity caused by antiviral intervention. Previously, the effect of antiviral therapy with NAs has been examined in general patients with chronic hepatitis B and T cell immunity was found to be restored while HBV DNA level decreased [28–30].

In this study, we examined the effects of antiviral intervention on maternal T cell immunity during pregnancy by comparing mothers who received antiviral intervention with those who did not, and investigate whether this effect is sustainable by comparing differences of T cells immunity between the intervention and non-intervention mothers after drug withdrawal postpartum.

## Materials and methods

### Study subjects

**Fig. 1:**
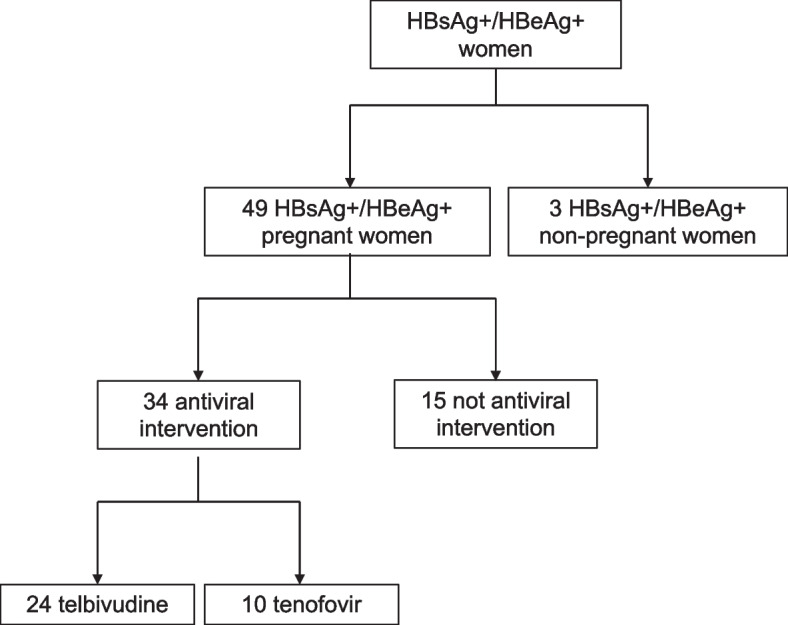
The Flowchart of subjects enrolment

We conducted a study to investigate the effects of maternal antiviral intervention on T cell immunity of HBV-infected mothers at delivery and postpartum. A total of 49 HBV-infected mothers were enrolled at delivery at the Nanfang Hospital, including 34 mothers who received prophylactic antiviral intervention from gestation weeks 26 to 28 until delivery to prevent MTCT (AVI group) and 15 of those who did not (NAVI group). In AVI group, 24 mothers received telbivudine (600 mg, once daily) and 10 received tenofovir disoproxil fumarate (300 mg, once daily). In addition, three HBsAg^+^/ HBeAg^+^ non-pregnant women were enrolled as control for comparison to pregnant status. Enrolment of study subjects was showed by the flowchart (Fig. 1). We followed up 20 intervention mothers and 6 non-intervention mothers at 6–8 and 15–18 weeks after postpartum, respectively. The main reason for loss to follow-up was moving back to hometown after delivery.

The criteria of enrolment of subject were HBeAg positivity, normal alanine aminotransferase (ALT), and high levels of HBV DNA (≥ 6 log_10_ IU/mL) before antiviral intervention on intervention group, and HBeAg positivity, normal ALT, high levels of HBV DNA (≥ 6 log_10_ IU/mL) at delivery on non-intervention group. All subjects recruited were negative for hepatitis C virus and human immunodeficiency virus. Additionally, those receiving concurrent treatment involving cytotoxic drugs, immune modulators, glucocorticoids, and with major systemic disease malignancies, liver cirrhosis, heart diseases, or renal dysfunctions were excluded. The subjects who met the criteria were enrolled consecutively in this study. This study was approved by the Ethics Committee of the Nanfang Hospital (NFEC-2019–197), and written informed consent was obtained from all subjects.

### Serological markers and HBV DNA assays

5 mL of venous blood was collected from each subject and serum was prepared for detection of serological markers and HBV DNA. The level of HBsAg [lower limit of detection (LOD): 0.05 IU/mL] and HBeAg or the presence of HBeAb (hepatitis B E antibody) was determined using a Roche Elecsys assay (Roche, Basel, Switzerland). Serum HBV DNA levels were quantitatively determined by real-time polymerase chain reaction using a commercial nucleic acid diagnostic kit (Sansure Biotech, Changsha, China) with an LOD of 100 IU/mL. Serological marker and HBV DNA assays were performed at the Laboratory of Viral Hepatitis Research, Nanfang Hospital. Serum ALT and AST were measured by rate method on an automatic biochemical analyzer (Beckman Coulter) at the Department of Clinical Laboratory, Nanfang Hospital.

### Preparation of peripheral blood mononuclear cells (PBMCs) and flow cytometric staining

PBMCs were isolated from 10 mL of anti-coagulation venous blood by Ficoll–Hypaque density gradient centrifugation and cryopreserved in liquid nitrogen for deferred analysis. Thawed PBMCs were stained with Live/Dead Fixable dead cell stain (eBioscience, San Diego, CA, USA). The cells were then stained for 30 min in the dark using the following antibodies (Abs): peridinin chlorophyll protein complex (PerCP)–Cy5.5- or fluorescein isothiocyanate (FITC)-conjugated anti-CD4 (clones SK3 and RAP-T4, respectively; BD Biosciences, San Jose, CA, USA), FITC- or allophycocyanin (APC)-conjugated anti-CD8 (clone RPA-T8; BD Biosciences), phycoerythrin (PE)-conjugated anti-CD45RA (HI100; BD Biosciences), PE–CY7-conjugated anti-CD62L (clone DREG-56; BD Biosciences), PE–CY7-conjugated anti-CD25 (clone M-A251; BD Biosciences), APC-conjugated anti-CD69 (clone FN50; Biolegend, San Diego, CA, USA), PE-conjugated anti-CTLA-4 (clone BNI3; BD Biosciences), PE-conjugated anti-PD-1 (clone MIH4; BD Biosciences), and PerCP–Cy5.5-conjugated anti-C-X-C chemokine receptor (CXCR)5 (clone J252D4; Biolegend). Intracellular APC-conjugated anti-forkhead box P3 (FOXP3; clone PCH101; eBioscience) staining was performed according to the manufacturer’s instructions. Stained cells were examined by flow cytometry using the BD FACSDiva system (BD Biosciences), and data were analysed using FlowJo software (TreeStar Inc., Ashland, OR, USA). The antibodies used in this analysis were listed in Table 1.

**Table 1:** Flow cytometry antibodies

Antibodies	Fluorescence	clones
Mouse Anti-Human CD4	FITC	RPA-T4
Mouse Anti-Human CD4	PerCP/Cyanine 5.5	SK3
Mouse Anti-Human CD8	FITC	RPA-T8
Mouse Anti-Human CD8	APC	RPA-T8
Mouse Anti-Human CD45RA	PE	HI100
Mouse Anti-Human CD62L	PECY7	DREG-56
Mouse Anti-Human CD25	PECY7	M-A251
Mouse Anti-Human CD69	APC	FN50
Mouse Anti-Human CTLA-4	PE	BNI3
Mouse Anti-Human PD-1	PE	MIH4
Mouse Anti-Human CXCR5	PerCP/Cyanine 5.5	J252D4
Rat Anti-Human Foxp3	APC	PCH101
Mouse Anti-Human IFN-γ	PECY7	B27
Rat Anti-Human IL10	PE	JES3-9D7
Mouse Anti-Human IL21	PE	3A3-N2.1
Mouse Anti-Human TNF-α	APC	6401.1111
Rat Anti-Human IL2	PE	MQ1-17H12
Rat Anti-Human IL4	PECY7	8D4-8

### In Vitro* Intracellular Cytokine Staining (ICS)*

For ICS, 5 × 10^5^ cells/well were stimulated with 5 μg/mL pre-coated anti-CD3 monoclonal Abs (mAb; clone 145-2C11; BD Biosciences) and 5 μg/mL soluble anti-CD28 mAbs (clone 37.51; BD Biosciences) in a 96-well flat-bottomed plate for 24 h. Brefeldin A (10 μg/mL; BD Biosciences) was added during the final 6 h of incubation. Following fixation and permeabilisation (BD Biosciences) for 20 min, surface-stained cells were incubated with the following antibodies for 30 min: PE–Cy7-conjugated anti-interferon (IFN)-γ (clone B27; BD Biosciences), PE-conjugated anti-interleukin (IL)-10 (clone JES3-9D7; Biolegend), PE-conjugated anti-IL-21 (clone 3A3-N2.1; BD Biosciences), APC-conjugated anti-tumour necrosis factor (TNF)-α (clone 6401.1111; BD Biosciences), PE-conjugated anti-IL-2 (clone MQ1-17H12; BD Biosciences), and PE–Cy7-conjugated anti-IL-4 (clone 8D4-8; BD Biosciences). The antibodies used in this analysis were listed in Table 1. Analyses were performed using the BD FACSDiva system (BD Biosciences) and the FlowJo software (TreeStar Inc.).

### Statistical analysis

Statistical analysis was performed using GraphPad Prism (v.8.0; GraphPad Software, San Diego, CA, USA), and data were expressed as either the median with a 95% confidence interval (CI) or an interquartile range. The non-parametric Mann–Whitney U, Kruskal–Wallis, and Fisher’s exact tests, Pearson’s correlation analysis or Spearman’s correlation analysis was used for statistical analyses, based on two-tailed hypothesis tests. *P* values < 0.05 were considered significant.

## Results

### Baseline characteristics of pregnant women with chronic HBV infection

To investigate the effects of prophylactic antiviral intervention on T cell immunity, 34 pregnant women in AVI group and 15 pregnant women in NAVI group were enrolled in this study. The baseline characteristics of pregnant women with chronic HBV infection in the two groups were showed in Table 2. There were no significant differences in age, gravidity, gestational age, and caesarean section rate between the two groups. The levels of ALT and AST in HBV-infected mothers in the AVI group or NAVI group were all within the normal range at delivery, and the median of ALT and AST (interquartile range) were 16.00 (12.50, 20.00) and 21.00 (17.00, 27.00) U/L, respectively, and no statistically significant differences were found in level of ALT and AST. We did not find significant difference in HBsAg and HBeAg level between the two groups. HBV DNA quantity in AVI group was significantly lower than that in the NAVI group (*p* < 0.001).

**Table 2:** Clinical characteristics of HBV-infected mothers at delivery

	**Intervention group (*n* = 34)**	**Non-intervention group (*n* = 15)**	***P* value**
Age-yr	27.50 (26.00,30.00)	26.00 (24.00,32.00)	0.535
Gravidity	1.00 (1.00,2.00)	1.00 (1.00,2.00)	0.774
Delivery by means of cesarean section — no. (%)	11 (32.35%)	5(33.33%)	> 0.999
Gestational age—wk	39.50 (39.00,40.00)	39.00 (38.00,39.00)	0.080
Duration of antiviral intervention—dy	89.50 (72.00, 100.0)	-	
LDT treatment-no.(%)	24(70.59%)	-	
TDF treatment-no.(%)	10(29.41%)	-	
ALT(U/L)	16.00(12.50,20.00)	20.00(15.00,35.00)	0.113
AST(U/L)	21.00(17.00,27.00)	24.00(16.00,30.00)	0.624
HBsAg(IU/ml)	15,549(3644,32,316)	8456(2229,52,000)	0.862
HBeAg(COI)	1320(809.6,1548)	1448(1174,1767)	0.219
HBV DNA(log_10_ IU/ml)	3.38 (2.38,4.37)	7.70 (6.58,8.57)	< 0.001

Data are shown as Median (IQR, interquartile range)

Abbreviations: *ALT* Alanine aminotransferase, *AST* Aspartate aminotransferase, *COI* cut off index, *IQR* Interquartile range, *LDT* Telbivudine, *TDF* Tenofovir disoproxil fumarate, *HBsAg* Hepatitis B surface antigen, *HBeAg* Hepatitis B E antigen, *HBV* hepatitis B virus, *DNA* Deoxyribonucleic acid

### Prophylactic antiviral intervention increased maternal Treg frequency at delivery

**Fig. 2:**
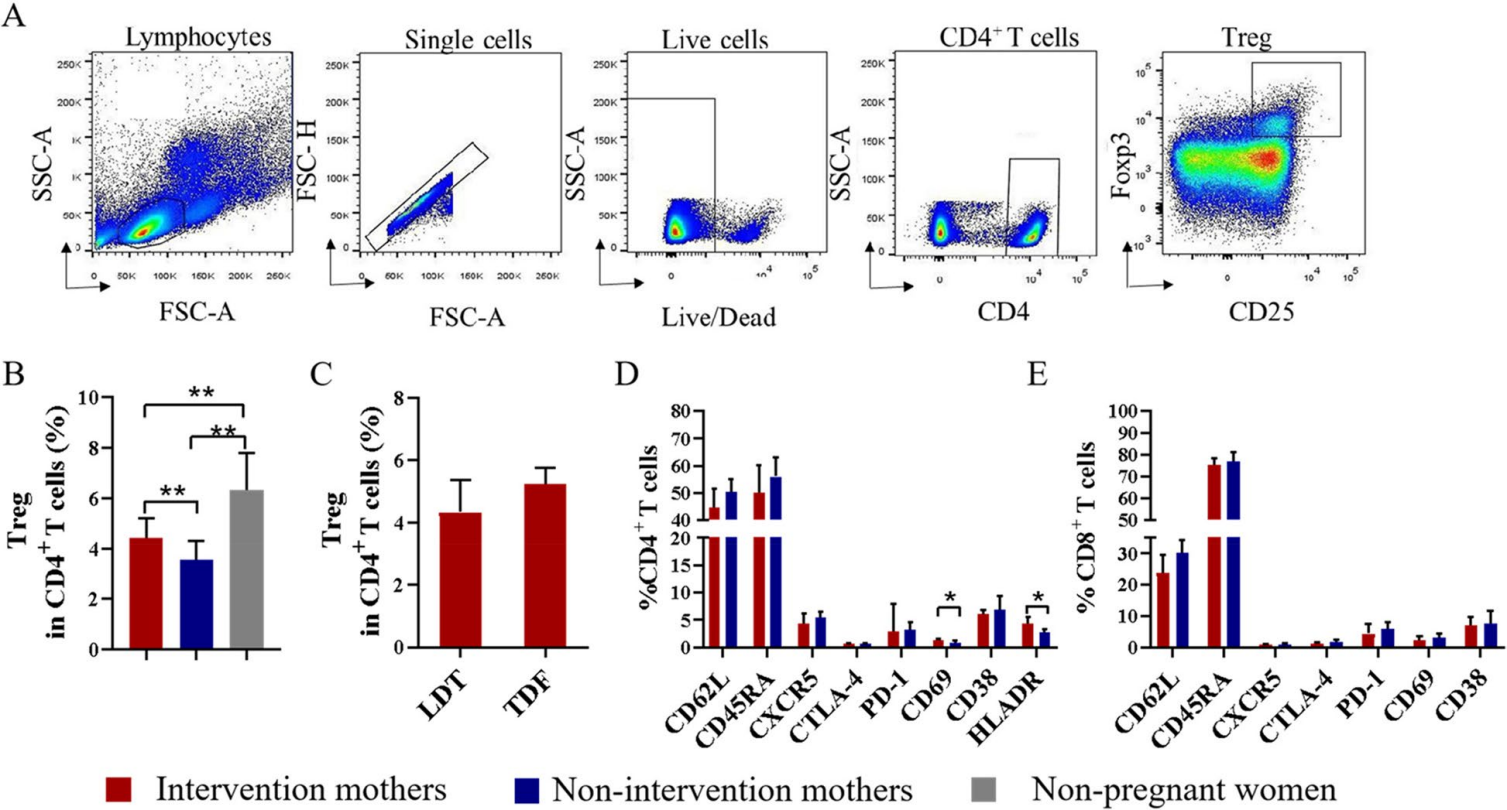
Prophylactic antiviral intervention increased maternal Treg frequency at delivery **A** Gating strategy for Tregs. PI was used in the Live/Dead Fixable dead cell stain kit to separate living cells. **B** Treg frequency in intervention mothers (*n* = 34), non-intervention mothers (*n* = 15), and HBsAg^+^ HBeAg^+^ non-pregnant women (IT, *n* = 3). **C** Treg frequency in mothers receiving LDT (*n* = 24) or TDF (*n* = 10) intervention. **D**-**E** Expression of CD62L, CD45RA, CXCR5, CTLA-4, PD-1, CD69, CD38, and HLADR on CD4^+^ T cells or CD8^+^ T cells (intervention mothers, *n* = 34; non-intervention mothers, *n* = 15). Data represent the median with 95% CI. *P < 0.05, **P < 0.005

Gating strategy for Treg is shown in Fig. 2A. Upon evaluating the effects of maternal antiviral intervention during pregnancy on T cell phenotypes, we found that Treg frequency in AVI group was significantly higher than that in NAVI group (*P* < 0.002). Furthermore, the frequency was significantly lower in AVI or NAVI mothers than in HBsAg^+^ HBeAg^+^ non-pregnant women (Fig. 2B). Among pregnant women in the AVI group, no significant difference in Treg frequency was noted among those with telbivudine (LDT, *n* = 24) or tenofovir (TDF, *n* = 10) intervention (*P* > 0.05) (Fig. 2C). In AVI group, activation-molecule expression in peripheral blood CD4^+^ T cells [CD69 and human leukocyte antigen–DR isotype (HLADR)] evidently increased (*P* < 0.05), but no similar expression was found in CD8^+^ T cells. Additionally, we observed no significant difference in the phenotypes (CD62L, CD45RA, CXCR5, CD38, CTLA-4, and PD-1) of CD4^+^ T cells or CD8^+^ T cells between AVI and NAVI groups (*P* > 0.05) (Fig. 2D and Fig. 2E). These results showed that maternal Treg frequency in pregnant women with HBV infection at delivery was lower than that of non-pregnant women with HBV infection and maternal antiviral intervention had effect of increasing maternal Treg frequency in pregnant women with HBV infection at delivery.

### Prophylactic antiviral intervention enhanced maternal Th2 response and suppressed Th1 response at delivery

**Fig. 3:**
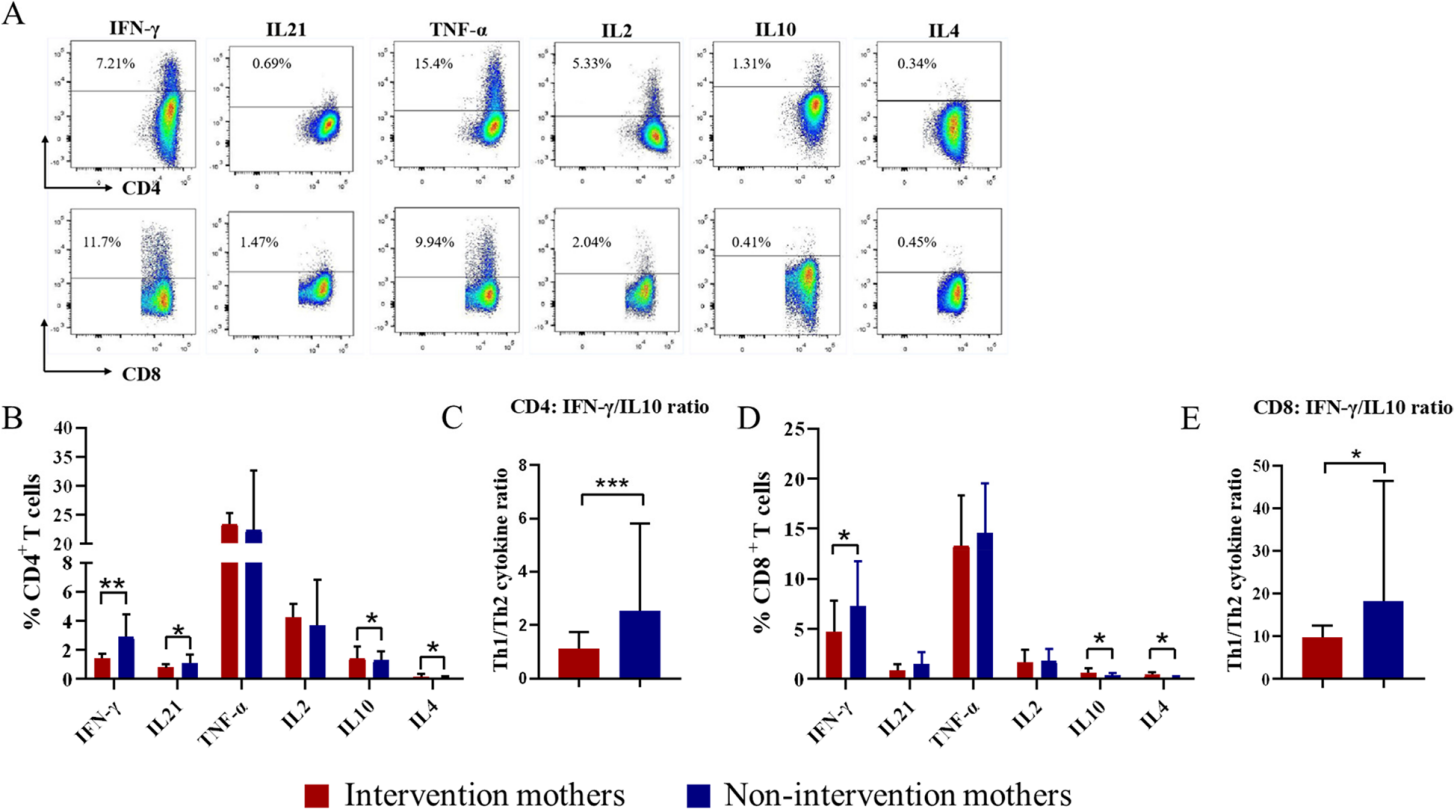
Prophylactic antiviral intervention enhanced maternal Th2 response and suppressed Th1 response at delivery PBMCs were stimulated with pre-coated anti-CD3 and soluble anti-CD28 antibodies for 24 h, with the addition of brefeldin A for the final 6 h. Cytokines production by T cells was measured using intracellular cytokine staining. (**A**) Representative dot plots of the production of Th1 cytokines (TNF-α, IFN-γ, and IL-2), Th2 cytokines (IL-4 and IL-10), and IL-21 in CD4^+^ or CD8^+^ T cells. (**B**) Percentages of cytokine-producing CD4^+^ T cells in intervention (*n* = 34) or non-intervention (*n* = 15) mothers, and (**C**) the data represent Th1:Th2-cytokine ratio (IFN-γ:IL-10) in CD4^+^ T cells. (**D**) Percentages of cytokine-producing CD8^+^ T cells in intervention (*n* = 34) or non-intervention (*n* = 15) mothers, and (**E**) the data represent the Th1:Th2-cytokine ratio (IFN-γ:IL-10) in CD8^+^ T cells. The data represent the median with 95% CI. *P < 0.05, **P < 0.005, ***P < 0.001

Following anti-CD3/anti-CD28 stimulation, we analysed the levels of cytokines secreted by CD4^+^ or CD8^+^ T cells in AVI group and NAVI group by flow cytometry. Representative FACS dot plots of cytokines production from T cells were shown in Fig. 3A. CD4^+^ T cells from AVI group displayed a reduced ability to secrete IFN-γ (*P* = 0.005) and IL-21 (*P* = 0.043), and an increased ability to secrete Th2-type cytokines (IL-10 and IL-4; *P* = 0.040 and *P* = 0.036, respectively), with the frequency ratio of IFN-γ-secreting CD4^+^ T cells to IL-10-secreting CD4^+^ T cells (Th1:Th2 ratio) in AVI group being significantly lower than that in NAVI group (*P* = 0.001) (Fig. 3B and Fig. 3C). Moreover, for CD8^+^ T cells, the AVI group displayed attenuated IFN-γ secretion (*P* = 0.028), but enhanced IL-10 and IL-4 secretion (*P* = 0.032 and *P* = 0.011) (Fig. 3D). Similarly, the ratio of IFN-γ-secreting CD8^+^ T cells to IL-10-secreting CD8^+^ T cells in the AVI group was significantly lower than that in NAVI group (*P* = 0.022) (Fig. 3E). There was no significant difference in the ability of CD4^+^ or CD8^+^ T cells to secrete TNF-α and IL-2 between the two groups.

### Treg frequency among intervention mothers correlated negatively with maternal HBsAg or HBeAg levels at delivery

**Fig. 4:**
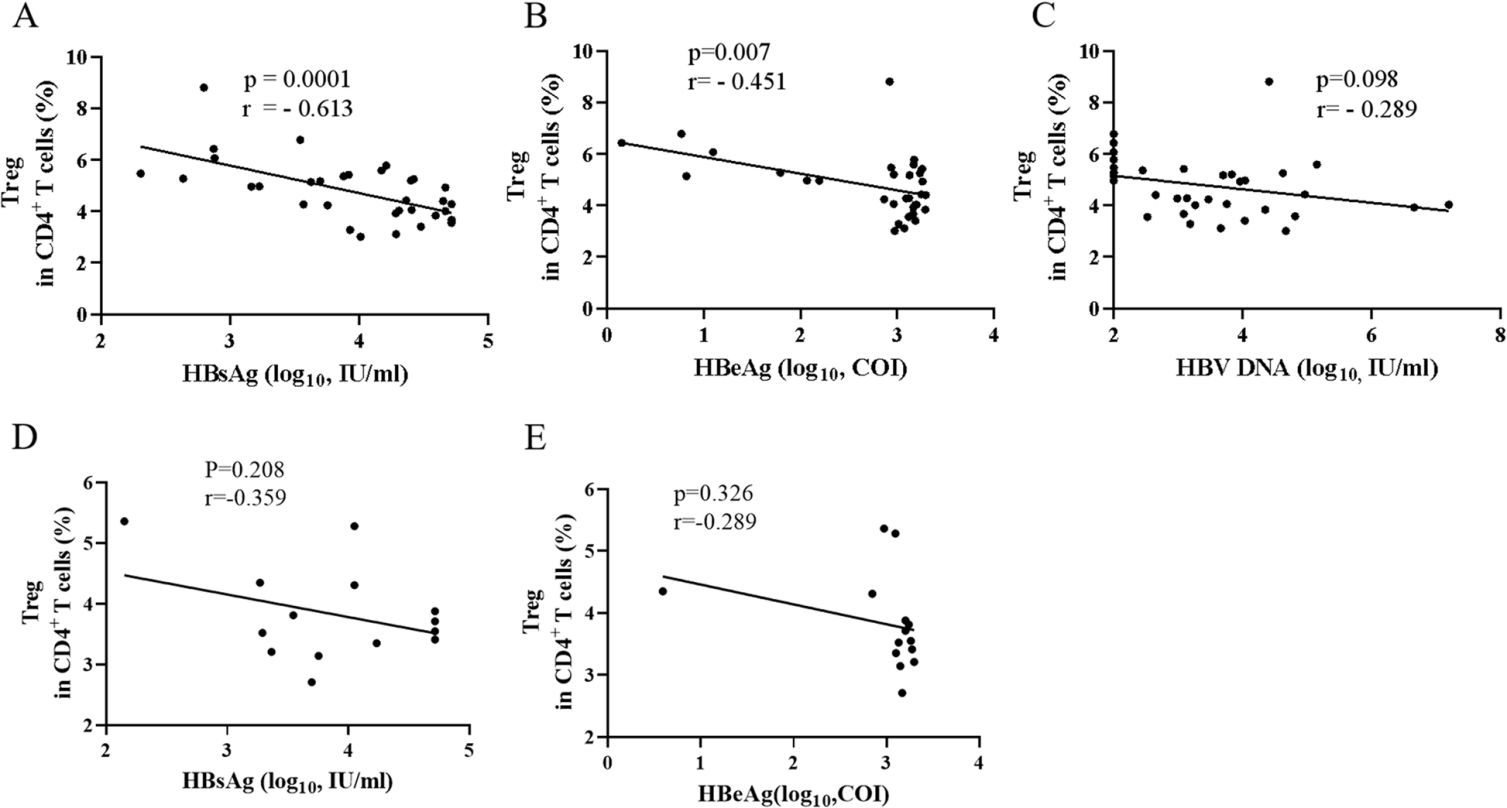
Correlation between Treg frequency and levels of maternal HBsAg, HBeAg, or HBV DNA in intervention mothers at delivery Correlation of Treg frequency with (**A**) HBsAg, (**B**) HBeAg, and (**C**) HBV DNA levels in intervention mothers (*n* = 34) and with (**D**) HBsAg and (**E**) HBeAg levels in non-intervention mothers (*n* = 15). r represents the correlation coefficient

Analysis of Treg frequency in AVI group and HBsAg, HBeAg, or HBV DNA revealed that Treg frequency was negatively correlated with maternal HBsAg or HBeAg levels (*P* < 0.001, r =  − 0.613; and *P* = 0.007, r =  − 0.451) (Fig. 4A and Fig. 4B). Additionally, we found no significant correlation between Treg frequency and maternal HBV DNA levels (*P* = 0.098, r =  − 0.289) (Fig. 4C). However, 15‒18 weeks after delivery, maternal HBeAg level was positively correlated with Treg frequency in AVI group (*P* = 0.034, r = 0.532), but a similar correlation was not observed between Treg frequency and HBsAg (see supplementary Fig.S1A and Fig.S1B). In NAVI group at delivery, no correlation was observed between Treg frequency and maternal HBsAg level (*P* = 0.208, r =  − 0.359) and HBeAg level (*P* = 0.326, r =  − 0.289) (see Fig. 4D and 4E).

### Prophylactic antiviral intervention during pregnancy had no significant effect on maternal T cells after delivery

**Fig. 5:**
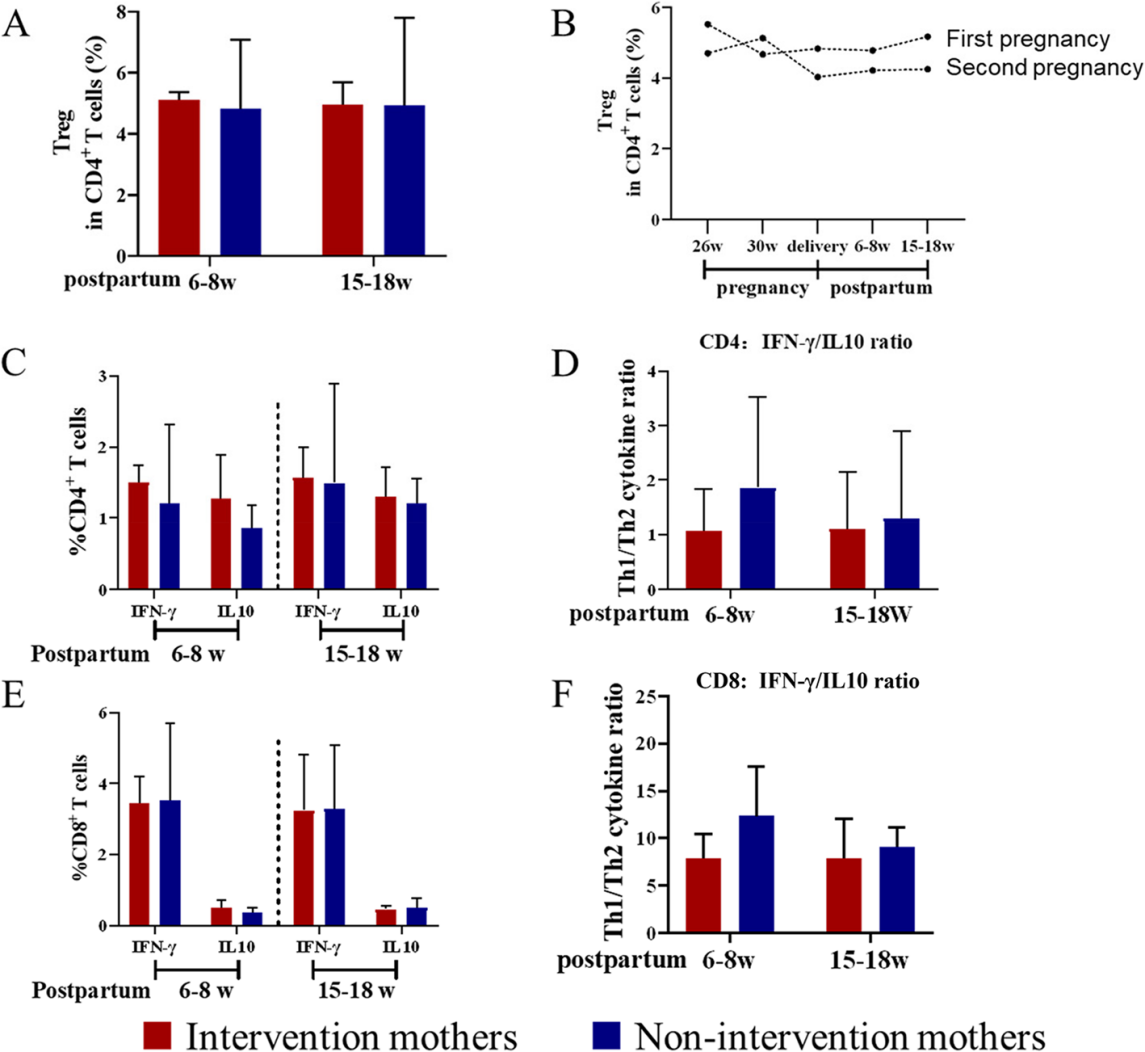
Prophylactic antiviral intervention in late pregnancy had no significant effect on maternal T cells after delivery (**A**) Comparison of the difference in Treg frequency in intervention mothers (*n* = 20) with non-intervention mothers (*n* = 6) at postpartum 6–8 weeks or 15–18 weeks; (**B**) Treg frequency changes before, during and after withdrawal of antiviral drugs in a chronic HBV infected mother during the first and second pregnancy; (**C**-**D**) Percentages of cytokine-producing CD4 + T cells in intervention (*n* = 20) or non-intervention (*n* = 6) mothers at postpartum 6–8 or 15–18 weeks, respectively and the data represent Th1:Th2-cytokine ratio (IFN-γ:IL-10) in CD4 + T cells. (**E–F**) Percentages of cytokine-producing CD8 + T cells in intervention (*n* = 20) or non-intervention (*n* = 6) mothers at postpartum 6–8 or 15–18 weeks, respectively and the data represent Th1:Th2-cytokine ratio ((IFN-γ:IL-10) in CD8 + T cells. The data represent the median with 95% CI. *P < 0.05

In comparing Treg frequency between AVI and NAVI group at 6–8 weeks postpartum or 15–18 weeks postpartum, we found no significant difference (Fig. 5A). In addition, we observed the changes in Treg frequency before and after the antiviral treatment of a mother with chronic HBV infection during the first and second pregnancy. We found that Treg frequency in the mother with the second baby was higher than with the first baby at one month after antiviral treatment, and at other follow-up time points, all lower than that with the first baby (Fig. 5B). The ability of CD4^+^ T cells or CD8^+^ T cells to secrete IFN-γ or IL-10 were also similar in these two groups after delivery. Furthermore, there was no significant difference in the ratio of IFN-γ-secreting T cells to IL-10-secreting T cells between two groups (Fig. 5C-5F).

## Discussion

It is widely accepted that antiviral intervention with NAs during pregnancy is a safe and effective measure for preventing MTCT. Currently, antepartum antiviral intervention has been recommended for preventing MTCT in the hepatitis B guidelines [18–21]. However, the safety issue for mothers has been questioned because ALT flares occur frequently in mothers who discontinued antiviral intervention postpartum [31–33]. Therefore, we investigated the effects of antiviral intervention during pregnancy on T cell immunity of chronic HBV-infected mothers.

Our results indicated that antiviral intervention during the third trimester increased maternal Treg frequency, enhanced Th2-type response and suppressed Th1-type response at delivery. We found a distinct profile of T cell immunity in patients receiving NAs relative to that observed in previous studies. Previous studies showed that treatment of CHB patients with NAs reduced Treg frequency and restored host immunity against HBV, which was associated with an upregulated Th1 response [30, 34, 35]. This disparity might be explained by the difference in the population cohort between ours and the reported studies. First, our study enrolled HBsAg^+^/HBeAg^+^women in the immunotolerance phase and not in the immune-active phase. Second, it has been shown that pregnant women exhibited immune-response profile distinct from that of non-pregnant patients due to changes in hormones and other components of the immune system during pregnancy [24, 25]. Third, the purpose of short-term NA administration in the present study was to prevent MTCT, and not to treat hepatitis B in CHB patients. Treg frequency and the abilities of cytokines secretion were similar between the AVI group and NAVI group after withdrawal treatment postpartum, and the second time antiviral intervention during another pregnancy had no superposition effect on Treg frequency, that is, the influence of the antiviral intervention in the third trimester on maternal T cells was not sustained after delivery.

Previous studies have showed that Th1 and Th2 responses during various stages of pregnancy are dynamically regulated, and during parturition, the last phase of pregnancy, the pro-inflammatory milieu, especially Th1 response, is predominant [25]. From our studies, we found that antiviral intervention enhanced maternal Th2 response and increased Treg frequency, which might change the pro-inflammatory milieu at delivery into relatively pro-inflammatory/anti-inflammatory balance. In a study on effect of telbivudine therapy on the cellular immune response in chronic hepatitis B, it was found that the decrease in serum HBV DNA levels was associated with an increase in IFN-γ and TNF-α production by HBV antigen-specific T cells. The difference of effect of antiviral therapy on Th1/Th2 immunity might be caused by the unique milieu of pregnancy which is characterized by immune tolerance to keep a successful pregnancy. This result indicated that high frequency of Treg in the intervention mothers suggests a relative balance between pro-inflammation and anti-inflammation.

Notably, we found a negative correlation between Treg frequency and maternal HBsAg or HBeAg in intervention mothers at delivery. Liu et al. [36] reported that Treg frequency in CHB patients under non-pregnancy conditions correlated positively with HBsAg, HBeAg and HBV DNA levels. This difference might be related to the effects of pregnancy, as we found a positive correlation between Treg frequency and HBeAg in intervention mothers after delivery. This result indicated that immunological characteristics of chronic HBV-infected women in pregnancy are distinct from that of non-pregnancy. Studies have shown that maternal peripheral blood in pregnancy had a reduced percentage of Treg compared with peripheral blood of non-pregnant women [37, 38], and our study also found similar reduction in maternal Treg frequency at delivery**.**It was reported that Tregs play an important role in maintaining immune tolerance in patients with chronic HBV infection [39, 40]. Furthermore, the extent of immunotolerance may be related to HBsAg or HBeAg levels. HBV-infected mothers with lower levels of HBsAg or HBeAg tend to break immunotolerance status. However, in the special milieu of pregnancy, the immune system of mother with lower levels of HBsAg or HBeAg requires higher Treg frequency to maintain immunotolerance, so that Treg frequency in the AVI group was negatively correlated with maternal HBsAg or HBeAg levels at delivery and positively correlated with maternal HBsAg or HBeAg levels after delivery.

There are some limitations in this study. First, the HBV-infected mothers were enrolled at delivery, thus, we could not collect PBMC samples before antiviral intervention. Therefore, we didn’t have the chance to study the change of T cell immunity before and after NAs initiation. Second, we don’t have non-pregnant HBV-infected women undergoing NAs treatment as control, because currently chronic HBV infection in the phase of immunotolerance is not indication for antiviral therapy according to hepatitis B guidelines. Third, we failed to detect the HBV-specific T lymphocyte subsets from the PBMC of HBV-infected mothers, probably due to lack of HBV-specific T lymphocytes in PBMC of HBV-infected mothers who are in the immunotolerance phase.

In summary, we found that prophylactic antiviral intervention during pregnancy increased maternal Treg frequency, enhanced Th2 response and suppressed Th1 response at delivery, which was not sustained after drug discontinuation postpartum. The findings in this study indicated that prophylactic antiviral intervention with NAs during pregnancy would not do harm to HBV-infected mothers in terms of liver function, because short-term NAs treatment tend to make T cell immunity of HBV-infected mothers more tolerance, with increased frequency of Treg and enhanced Th2-type response.

In the future, more studies are needed to explore dynamic change of T cell immunity over pregnancy and postpartum and it’s relationship to progression of hepatitis B in pregnant women with chronic hepatitis B.

## Supplementary Information

**Additional file 1: SupplementaryFigures.  **Correlation between Treg frequency and levels of maternal HBsAg and HBeAg in intervention mothers at 15‒18 weeks postpartum.

## Data Availability

The data that support the findings of this study are available from the corresponding author upon reasonable request.

## Abbreviations

AASLD: American Association for the study of Liver Diseases
Abs: Antibodies
APASL: Asian Pacific Association for the Study of the Liver
AVI: Antiviral intervention
EASL: European Association for the Study of the Liver
CTLA: Cytotoxic T-lymphocyte-associated antigen
HBV: Hepatitis B virus
HBsAg: Hepatitis B surface antigen
HBeAg: Hepatitis B E antigen
MTCT: Mother-to-child transmission
NAs: Nucleot(s)ide analogues
NAVI: Non-antiviral intervention
PBMCs: Peripheral blood mononuclear cells
Th: T helper cell
Treg: Regulatory T cell
