# Supplementary material for: Effect of prophylactic antiviral intervention on T cell immunity in hepatitis B virus-infected pregnant women
Source: BMC Pregnancy Childbirth. 2023 May 27;23:392. doi: 10.1186/s12884-023-05700-8 (PMC10223933; doi:10.1186/s12884-023-05700-8)
Supplement: Supplementary file 1 — Additional file 1: SupplementaryFigures. Correlation between Treg frequency and levels of maternal HBsAg and HBeAg in intervention mothers at 15‒18 weeks postpartum. [file 12884_2023_5700_MOESM1_ESM.pptx]

## Slide 1
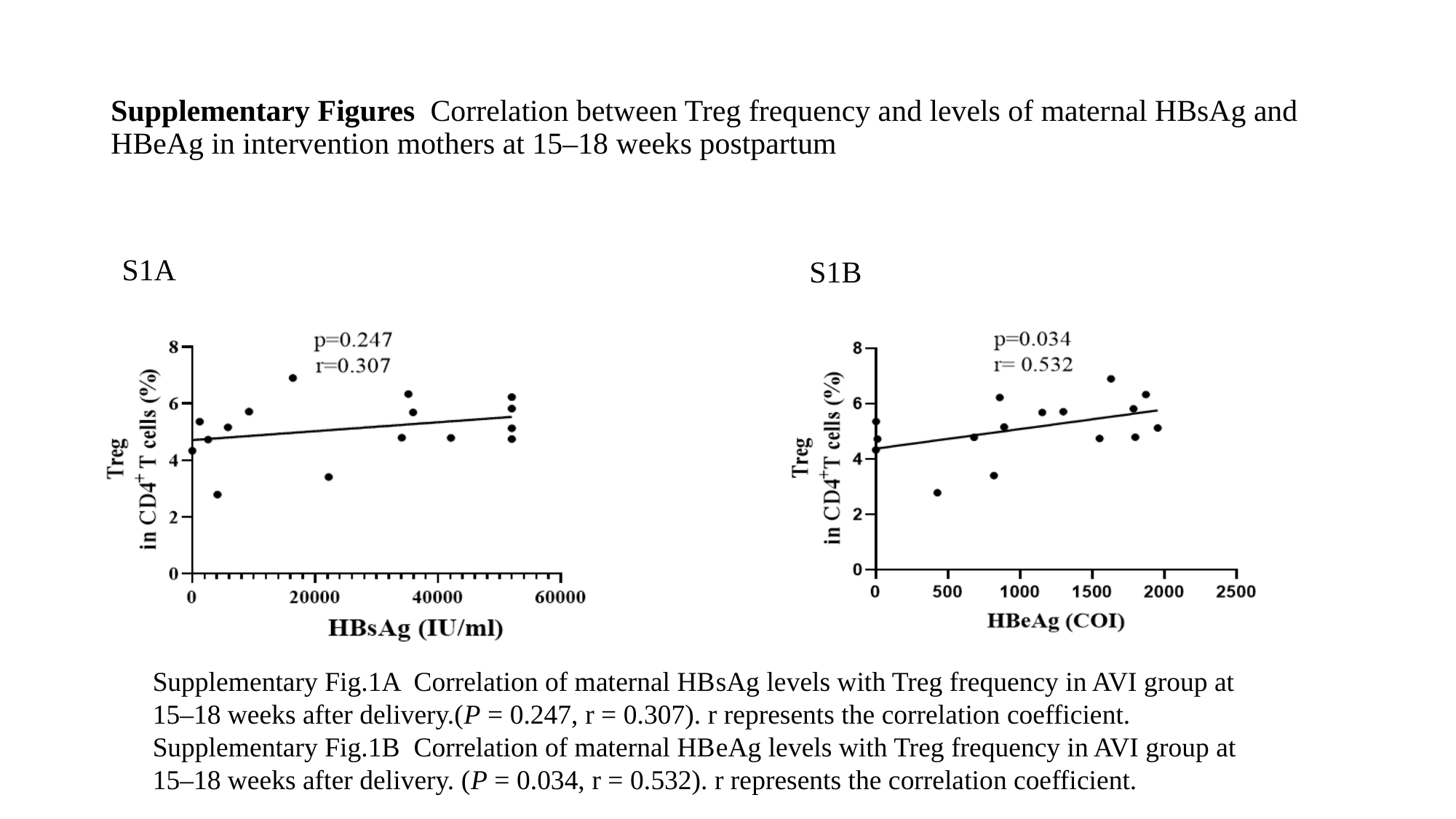

# Supplementary Figures Correlation between Treg frequency and levels of maternal HBsAg and HBeAg in intervention mothers at 15‒18 weeks postpartum
S1A
S1B
Supplementary Fig.1A Correlation of maternal HBsAg levels with Treg frequency in AVI group at 15‒18 weeks after delivery.(P = 0.247, r = 0.307). r represents the correlation coefficient.
Supplementary Fig.1B Correlation of maternal HBeAg levels with Treg frequency in AVI group at 15‒18 weeks after delivery. (P = 0.034, r = 0.532). r represents the correlation coefficient.
